# Prognostic effects of delirium motor subtypes in hospitalized older adults: A prospective cohort study

**DOI:** 10.1371/journal.pone.0191092

**Published:** 2018-01-30

**Authors:** Thiago Junqueira Avelino-Silva, Flavia Campora, Jose Antonio Esper Curiati, Wilson Jacob-Filho

**Affiliations:** Division of Geriatrics, Department of Internal Medicine, University of Sao Paulo Medical School, Sao Paulo, Sao Paulo, Brazil; University of Glasgow, UNITED KINGDOM

## Abstract

**Objectives:**

To investigate the association between delirium motor subtypes and hospital mortality and 12-month mortality in hospitalized older adults.

**Design:**

Prospective cohort study conducted from 2009 to 2015.

**Setting:**

Geriatric ward of a university hospital in Sao Paulo, Brazil.

**Participants:**

We included 1,409 consecutive admissions of acutely ill patients aged 60 years and over. We excluded admissions for end-of-life care, with missing data on the main variables, length of stay shorter than 48 hours, or when consent to participate was not given.

**Main outcomes and measures:**

Delirium was detected using the Confusion Assessment Method and categorized in hypoactive, hyperactive, or mixed delirium. Primary outcomes were time to death in the hospital, and time to death in 12 months (for the discharged sample). Comprehensive geriatric assessment was performed at admission and included socio-demographic, clinical, functional, cognitive, and laboratory variables. Further clinical data were documented upon death or discharge. Multivariate analyses used Cox proportional hazards models adjusted for possible confounders.

**Results:**

We included 1,409 admissions, with a mean age of 80 years. The proportion of in-hospital deaths was 19%, with a cumulative mortality of 38% in 12 months. Delirium occurred in 47% of the admissions. Hypoactive delirium was the predominant motor subtype (53%), followed by mixed delirium (30%) and hyperactive delirium (17%). Hospital mortality rates were respectively 33%, 34% and 15%. We verified that hypoactive and mixed delirium were independently associated with hospital mortality, with respective hazard ratios of 2.43 (95%CI = 1.64–3.59) and 2.31 (95%CI = 1.53–3.50). Delirium motor subtypes were not independently predictive of 12-month mortality.

**Conclusions:**

One in three acutely ill hospitalized older adults who suffered hypoactive or mixed delirium died in the hospital. Clinicians should be aware that hypoactive symptoms of delirium, whether shown exclusively or in alternation with hyperactive symptoms, are indicative of a worse prognosis in this population.

## Introduction

Delirium affects up to 50% of hospitalized older adults. It is associated with several unfavorable outcomes and it is estimated that more than US$160 billion are annually spent on the disorder in the United States [1–3]. It typically manifests with either of two patterns of psychomotor activity: hypoactive or hyperactive [4]. Hypoactive delirium is characterized by stupor, psychomotor lentification and lethargy, and is the most frequent pattern in older adults [5]. Conversely, hyperactive delirium is characterized by agitation, hypervigilance and hallucinations. Cases in which an alternance of the two patterns is observed are common and classified as mixed delirium [6].

The more exuberant hyperactive delirium is easier to detect and is often associated with alcohol withdrawal [7]. Hypoactive delirium frequently goes undetected or is interpreted as a mood disorder or fatigue, which can delay diagnosis and the implementation of therapeutic measures [8, 9]. Although the hypoactive pattern is more commonly indicated in the literature as being associated with a worse prognosis, current evidence to support this claim is conflicting. Of the seven studies identified in a recent systematic review that examined the effects of motor subtype on the clinical course of patients with delirium, only three showed an association between hypoactive delirium and a worse prognosis [10–13]. Of the four other studies, one concluded that hyperactive delirium had worse prognosis [14], and the other three did not find any association between delirium motor subtypes and the examined outcomes [15–17].

The inconsistency in findings concerning delirium motor subtypes and prognosis has important practical implications, both in the clinical and in the research context. Clinician education and awareness to the meaning of different patterns of psychomotor activity depend on these results. Likewise, researchers working on the evaluation of delirium severity need reliable data to adequately incorporate psychomotor activity in their assessment tools. For example, commonly used scales such as the Delirium Rating Scale and the Memorial Delirium Assessment Scale tend to value symptoms typical of hyperactive delirium, despite allusions to hypoactive delirium as potentially more severe [18, 19].

Therefore, we proposed to add to previous studies examining the association between delirium motor subtypes and mortality by following a large, consecutive, well characterized sample, and adjusting our analyses to several important covariates. In this study, we sought to investigate the effects of delirium motor subtypes on hospital mortality and 12-month mortality of acutely ill older adults.

## Methods

### Study design and population

This was a prospective cohort study involving acutely ill patients admitted to a geriatric ward of a 2,200-bed tertiary university hospital in Sao Paulo, Brazil. This 24-bed unit admits medical patients aged 60 years and over, and is staffed with a multidisciplinary team that includes geriatricians, nurses, physiotherapists, speech therapists, social workers, psychologists and nutritionists. Despite age being the only criterion for admittance in the ward, as a specialty unit with a limited number of beds, older adults with high clinical complexity and vulnerability are preferably referred for admission.

We included consecutive hospitalizations from January 2009 to June 2015. Inclusion criteria for the study were: (1) age of 60 years or over; (2) admission for acute illness (defined as disease of recent onset, or as recent complication of chronic disease, requiring hospitalization for clinical management). Exclusion criteria included: (1) admission for end-of-life care; (2) incomplete data on the main variables; (2) length of stay shorter than 48 hours; (4) patient or caregiver refusal to authorize use of hospital data for research.

### Measures

#### Outcomes

The primary endpoints for the study were: (1) time to death during hospitalization, analyzed for the whole sample; (2) time to death in 12 months, analyzed for participants who were discharged from the hospital.

Time to death in the hospital was documented at the end of hospitalization. Time to death in the follow-up period was obtained by TJAS, who attempted to contact patients 12 months after admission, in up to three telephone contacts. Dates of death were provided by family members, who were asked to confirm the information in their copies of the death certificates. When unable to reach patients or caregivers, TJAS examined medical records (including medical consultations, consultations with multidisciplinary team, records of tests and procedures) to identify information regarding dates of death, or the last dates on which the participants were known to be alive. When verifiable information was limited to the later, cases were considered lost to follow-up.

#### Predictors

Patients were evaluated according to a standardized comprehensive geriatric assessment that is routinely performed within the first 24 hours of admission [20]. Additional information regarding the characteristics of delirium (time of onset, predominant motor subtype, primary precipitating factors), new diagnoses and complications, functional status, and hospitalization summary, were recorded at discharge or death. Trained geriatric fellows performed the assessments under the supervision of experienced geriatricians. Study data were collected and managed using REDCap electronic data capture tools [21].

Delirium was detected using the Short Confusion Assessment Method (Short CAM) [22], both at admission and throughout the hospital stay. The CAM has been successfully validated against different editions of the Diagnostic and Statistical Manual for Mental Disorders, and has been reported as the most widely used standardized instrument to detect delirium in clinical practice and research [23], Delirium severity was measured using the Delirium Index, which assesses seven domains of the Long CAM (attention, thinking, level of consciousness, orientation, memory, sensory perception, and psychomotor activity) and generates scores ranging from zero to 21 (21 = most severe) [24], The primary independent variable was delirium motor subtype, which was defined according to predominant psychomotor features as hyperactive, hypoactive or mixed. This classification was completed upon discharge or death by the fellow and attending physician responsible for each case, and was based on the clinical features presented throughout the hospital stay. Characteristics of psychomotor agitation (voluntary or involuntary increased level of motor activity, such as restlessness, picking at bedclothes, tapping fingers, making frequent or sudden changes of position) and/or retardation (voluntary or involuntary sluggishness, staring into space, staying in one position for a long time, or moving very slowly) were observed for during the daily clinical follow-up and were established using the CAM Training Manual as reference [25].

Covariates of interest included: sociodemographic information (age; sex; self-reported race; marital status; literacy; economic classification; referring unit); medical history and physical examination data (visual and auditory deficits; vital signs; admission diagnoses); comorbidities (Charlson Comorbidity Index) [26]; cognitive status (Clinical Dementia Rating, CDR; Short Form of the Informant Questionnaire on Cognitive Decline in the Elderly, Short IQCODE) [27, 28]; functional status (six activities of daily living, ADLs; each activity was scored on a scale ranging from zero to two points and a final score was generated from the total sum of the items; range = 0–12, 12 = best) [29]; nutritional status (Mini Nutritional Assessment, MNA) [30]; polypharmacy (defined as the chronic use of five or more medications) [31]; laboratory tests (hemoglobin; total leukocytes; C-reactive protein; glomerular filtration rate, GFR; urea; sodium; potassium; sodium bicarbonate; albumin; 25-hydroxy vitamin D). To characterize baseline cognitive status and separate it from acute impairment, informants were instructed to respond to CDR and Short IQCODE items using conditions at 3 moths before admission as reference for baseline status [20].

### Statistical analysis

A descriptive analysis of demographic, clinical and laboratory characteristics, was performed using counts and proportions, means and standard deviations, medians and interquartile ranges. Categorical variables were compared across the study groups (no delirium, hyperactive, mixed, and hypoactive delirium) using Chi-squared test or Fisher’s exact test as appropriate. Continuous variables were compared using one-way ANOVA or Kruskal Wallis test as appropriate.

Kaplan Meier curves were used to represent unadjusted hospital survival and 12-month survival according to delirium motor subtypes, and log-rank tests to compare the groups. The associations between delirium motor subtypes and time to death were analyzed using Cox proportional hazards models, with “no delirium” as the referent category and adjusted to the following pre-selected covariates: age; sex; marital status; referring unit; ADL dependency level; dementia diagnosis and severity; nutritional status; comorbidities (hypertension, diabetes, heart failure, cerebrovascular disease, coronary disease, chronic obstructive pulmonary disease, cancer); polypharmacy; vital signs (heart rate, mean arteria pressure); GFR; urea; albumin; total leucocytes; C-reactive protein. The analyses were performed within clusters of patients, considering each individual might have had more than one hospitalization throughout the years. We used cluster-analysis routines which specify that the standard errors allow for intragroup correlations, adjusting the model to the fact that observations are independent across clusters, but not necessarily within. Since the purpose for these procedures was to group hospitalizations from the same patient, we employed constraint-based clustering to define groups based on medical record identifications. All statistical tests were two-tailed and an alpha error of up to 5% was accepted. Statistical analyses were performed using Stata SE 14.1 (Stata Corp, College Station, TX).

### Ethical considerations

This study was approved by the local institutional review board (Comissao de Etica para Analise de Projetos de Pesquisa do Hospital das Clinicas da Faculdade de Medicina de Universidade Sao Paulo). Written informed consent was obtained from all cases according to the principles expressed in the Declaration of Helsinki. Capacity to consent was based on cognitive assessments as described above, and determined according to the ability to clearly communicate decisions and understand relevant clinical information [32]. Dual consent was obtained from participants and their legal surrogates when mild to moderate cognitive decline was detected, and only from the surrogates when moderate to severe decline was present.

All patient identifiable information was stored in locked cabinets and/or secure electronic servers.

## Results

### Overall characteristics

**Fig 1:**
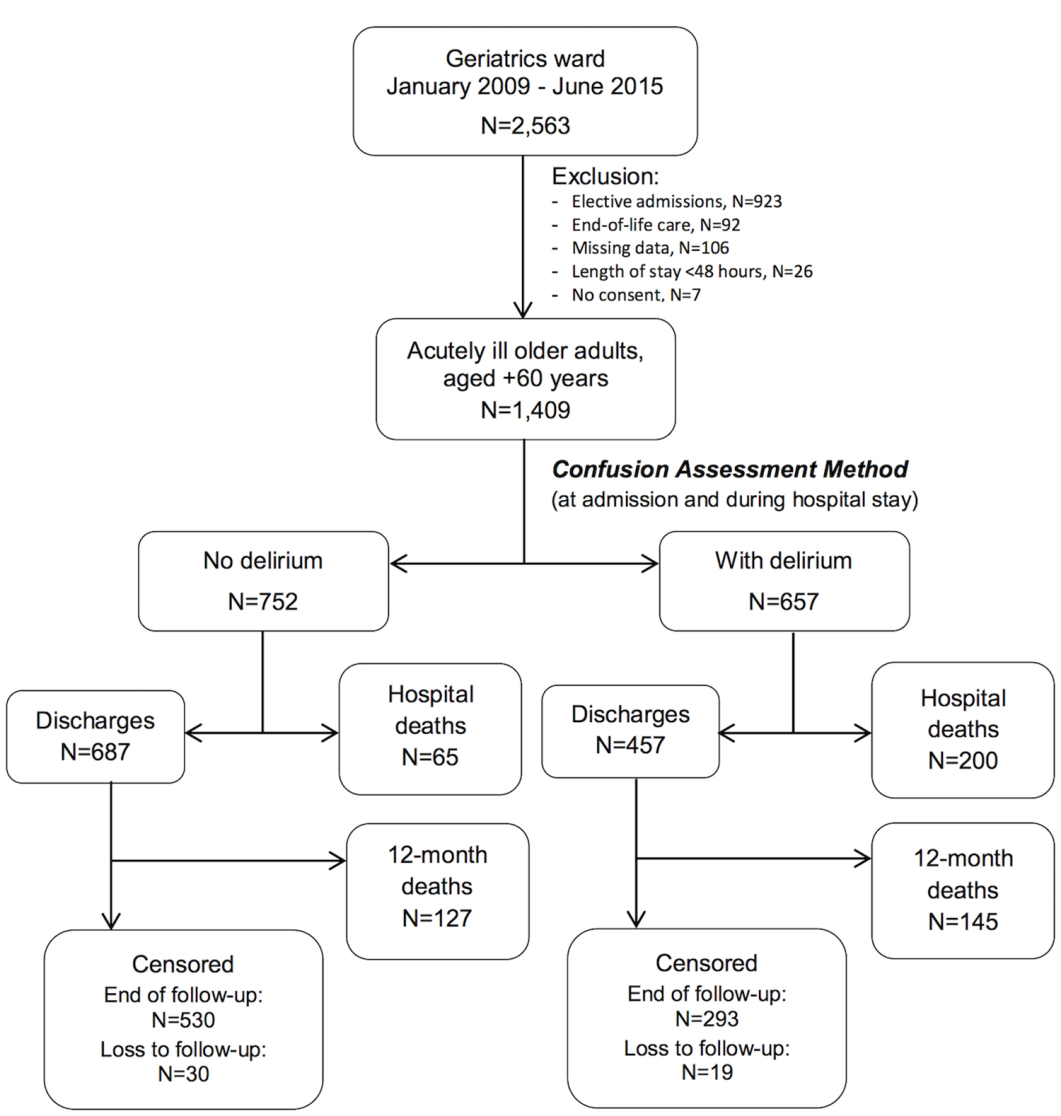
Flowchart of inclusion and follow-up in the study. A total of 1,409 admissions from 2009 to 2015 were included in the study, with 657 cases of delirium. Hypoactive delirium was identified in 348 admissions, followed by mixed delirium (197) and hyperactive delirium (112). Missing data represented only a minor proportion of the sample and was excluded from the analysis.

There were 1,409 hospitalizations that met inclusion and exclusion criteria, representing 1,204 clusters of patients (Fig 1). Participants were predominantly aged 80 years and over, female and from middle to low income groups (Table 1). Most admissions were referred from the emergency department (ED) and nearly half of these cases waited at least 48 hours before being transferred to our unit. Median length of hospital stay was of 15 days (interquartile range, IQR = 9;26). The median Charlson Comorbity Index score was 3 (IQR = 1;5). Infectious diseases were common at admission (48%), the most prevalent of which were pneumonia (41%) and urinary tract infection (34%).

**Table 1:** Characteristics of acutely ill hospitalized older adults, according to delirium motor subtype; 2009–2015*.

Characteristics, N (%)	Total 1409 (100)	No delirium 752 (53)	Hyperactive delirium 112 (8)	Mixed delirium 197 (14)	Hypoactive delirium 348 (25)	p-value
**Demographics**						
Age (years), mean (SD)	80 (±9)	79 (±9)	81 (±8)	83 (±9)	83 (±9)	<.001
60–69	175 (12)	127 (17)	9 (8)	15 (8)	24 (7)	<.001
70–79	440 (31)	264 (35)	38 (34)	49 (25)	89 (26)	
80–89	558 (40)	267 (36)	45 (40)	81 (41)	165 (47)	
≥90	236 (17)	94 (13)	20 (18)	52 (26)	70 (20)	
Female	860 (61)	451 (60)	57 (51)	116 (59)	236 (68)	.006
Marital status						
Married	539 (38)	313 (41)	51 (46)	66 (34)	109 (31)	<.001
Widowed	657 (47)	322 (43)	40 (36)	101 (51)	194 (56)	
Single	124 (9)	62 (8)	18 (16)	15 (8)	29 (8)	
Divorced/ separated	89 (6)	55 (7)	3 (3)	15 (8)	16 (5)	
Referring unit						
Outpatient clinics	536 (38)	358 (48)	24 (21)	58 (29)	96 (28)	<.001
Emergency department	772 (55)	351 (47)	72 (64)	128 (65)	221 (64)	
Intensive care	101 (7)	43 (6)	16 (14)	11 (6)	31 (9)	
**Geriatric Assessment**						
Polypharmacy	851 (60)	467 (62)	65 (58)	109 (55)	210 (60)	.349
Admission ADLs (points)						
9–12	485 (34)	402 (53)	22 (20)	26 (13)	35 (10)	<.001
5–8	315 (22)	187 (25)	24 (21)	36 (18)	68 (20)	
0–4	609 (43)	163 (22)	66 (59)	135 (69)	245 (70)	
Depression	313 (22)	162 (22)	15 (15)	52 (26)	82 (24)	.124
Dementia						
Absent	722 (51)	504 (67)	41 (37)	75 (38)	102 (29)	<.001
Mild	312 (22)	142 (19)	24 (21)	59 (30)	87 (25)	
Moderate	171 (12)	56 (7)	25 (22)	25 (13)	65 (19)	
Severe	204 (14)	50 (7)	22 (20)	38 (19)	94 (27)	
Mini Nutritional Assessment						
Normal	213 (15)	166 (22)	13 (12)	13 (7)	21 (6)	<.001
Risk of malnutrition	595 (42)	362 (48)	58 (52)	70 (36)	105 (30)	
Malnutrition	601 (43)	224 (30)	41 (37)	114 (58)	222 (64)	
**Comorbidities**						
Hypertension	1020 (72)	546 (73)	85 (76)	141 (72)	248 (71)	.803
Diabetes	448 (32)	229 (30)	39 (35)	68 (35)	112 (32)	.616
Heart failure	389 (28)	216 (29)	28 (25)	59 (30)	86 (25)	.415
Previous stroke	286 (20)	123 (16)	24 (21)	46 (23)	93 (27)	.001
Coronary disease	246 (17)	138 (18)	15 (13)	32 (16)	61 (18)	.593
COPD	172 (12)	100 (13)	12 (11)	20 (10)	40 (11)	.571
Cancer	154 (11)	86 (11)	10 (9)	23 (12)	35 (10)	.791
**Vital signs**						
Heart rate (bpm)						
<60	61 (4)	38 (5)	5 (5)	5 (3)	13 (4)	.434
60–100	1248 (89)	669 (89)	99 (88)	174 (88)	306 (88)	
≥100	100 (7)	45 (6)	8 (7)	18 (9)	29 (8)	
Mean arterial pressure <90mmHg	719 (51)	380 (51)	52 (46)	106 (54)	181 (52)	.622
**Laboratory tests**						
Leucocytes ≥11*10^3^ cells/mm^3^	321 (23)	143 (19)	26 (23)	60 (30)	92 (26)	.002
Albumin <3.3 g/dL	843 (60)	393 (52)	77 (69)	126 (64)	247 (71)	<.001
GFR (mL/min)						
≥60	705 (50)	382 (51)	70 (63)	89 (45)	164 (47)	<.001
30–59	492 (35)	275 (36)	28 (25)	59 (30)	130 (37)	
<30	212 (15)	95 (13)	14 (13)	49 (25)	54 (16)	
Urea ≥85 mg/dL	328 (23)	149 (20)	17 (15)	63 (32)	99 (28)	<.001
C-reactive protein >50 mg/L	560 (40)	254 (34)	51 (46)	94 (48)	161 (46)	<.001

SD = standard deviations; ADLs = activities of daily living; COPD = chronic obstructive pulmonary disease; bpm = beats per minute; GFR = glomerular filtration rate.

* Categorical variables were compared across the study groups using Chi-squared test or Fisher’s exact test as appropriate. Continuous variables were compared using one-way ANOVA or Kruskal Wallis test as appropriate. Complete descriptive data can be found in the supplemental material (S1–S3 Tables).

One in four admissions were of patients with moderate to severe dementia. Alzheimer's disease corresponded to 38% of the cases of dementia, while vascular dementia was observed in 23% of cases, mixed dementia in 20%, and other causes in 19%. The median score in the Short IQCODE was 3.63 (IQR = 3.13;4.56). Only 15% of the patients could be classified as having a normal nutritional status according to the MNA, which had a median score of 8 (IQR = 5;11). Other common geriatric syndromes in our sample were depression, urinary incontinence, falls, sensory deficits, and pressure ulcers. In the initial laboratory assessment, the mean value of serum albumin was low (3.2 ±0.6 g/dL) and the median value of C-reactive protein was high (37 ng/L, IQR = 11; 88). Mean hemoglobin level at admission was 11 (±2.3) g/dL and mean GFR was 67 (±39) mL/min.

### Delirium characteristics

We identified delirium at admission in 379 (27%) hospitalizations (Table 1). We subsequently detected delirium in the course of an additional 278 (20%) hospitalizations. Patients with delirium had a mean age of 83 (±8) years and 62% were women. When compared to patients without delirium, they were older (p <.001) and more often admitted from the emergency and intensive care units (p <.001). When compared to participants without delirium, those who suffered from the condition had a greater proportion of ADL dependency (p <.001), dementia (p <.001) and malnutrition (p <.001). Hypoactive delirium was the most frequently observed motor subtype in our setting (53%), followed by mixed delirium (30%) and hyperactive delirium (17%). The median Delirium Index score was 13 (IQR = 9;16), which is suggestive of moderate delirium symptom severity, and we did not find differences in scores between delirium motor subtypes.

### Unadjusted mortality

**Fig 2:**
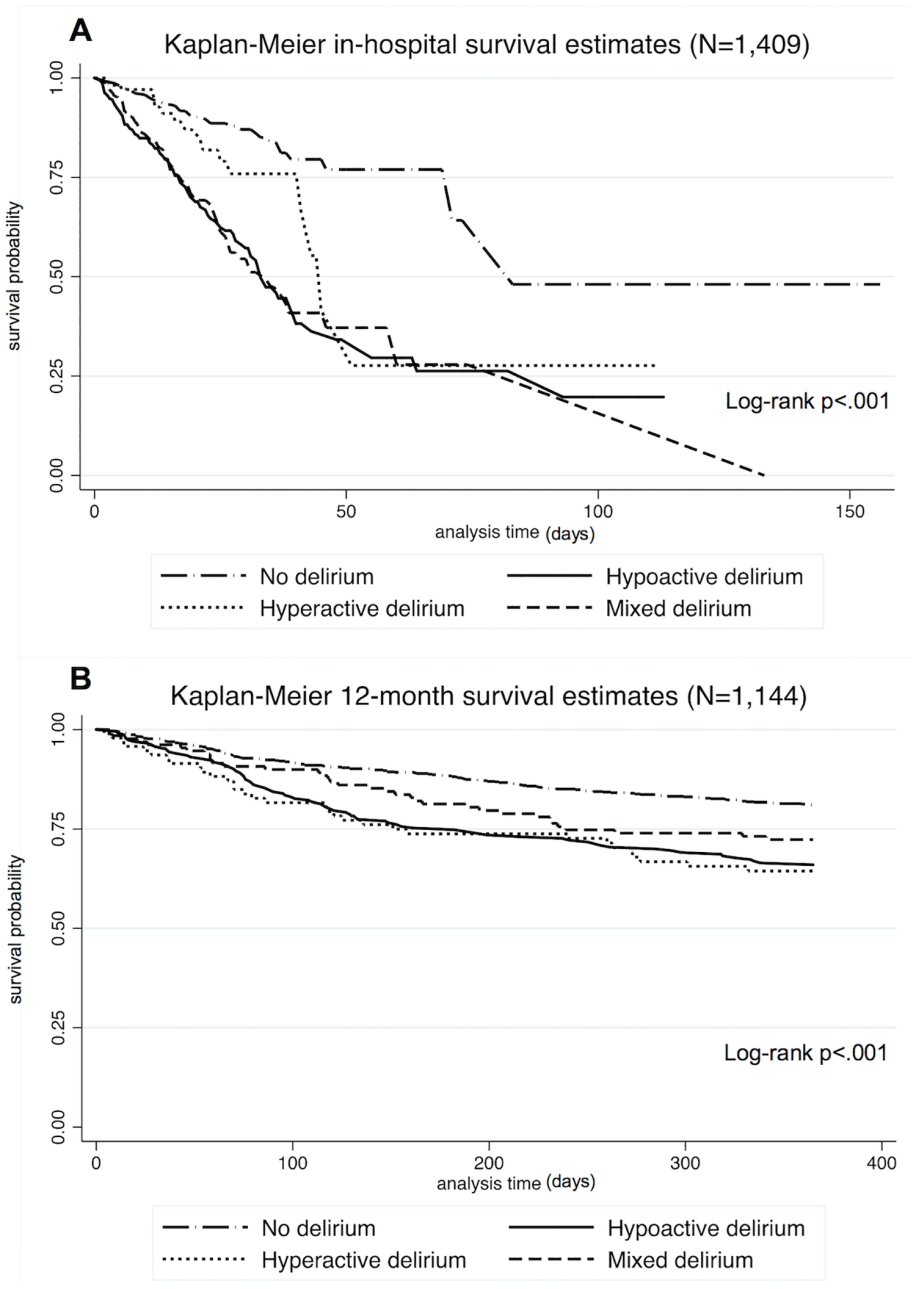
Probability of survival according to delirium motor subtypes. A. Kaplan-Meier estimates representing the probability of survival in the hospital according to delirium motor subtypes, with a corresponding log-rank test indicating a statiscally significant difference between the groups; B. Kaplan-Meier estimates representing the probability of survival in 12 months according to delirium motor subtypes, with a corresponding log-rank test indicating a statiscally significant difference between the groups.

A total of 265 (19%) older adults died in the hospital and an additional 272 died in the follow-up period after discharge. In-hospital mortality reached 30% in the delirium group, but was of only 9% in the non-delirious group (Pearson *X*^2^ = 109; p <.001). After discharge, 12-month mortality reached 32% in the delirium group, compared to 18% in the group without delirium (Pearson *X*^2^ = 27; p <.001). We found the following hospital mortality rates according to delirium motor subtypes: 15% for hyperactive delirium; 34% for mixed delirium; and 33% for hypoactive delirium. After discharge, the proportion of 12-month deaths for hyperactive, mixed and hypoactive delirium were, respectively, 34%, 27% and 34%. The probabilities of survival differed according to delirium motor subtype and are represented in Fig 2.

### Effects of delirium motor subtypes on mortality

We verified an independent association between hypoactive and mixed delirium subtypes and hospital mortality in a model adjusted for several possible confounders (age, sex, marital status, referring unit, functional status, cognitive status, nutritional status, comorbidities, polypharmacy, vital signs, glomerular filtration rate, urea, albumin, total leucocytes, C-reactive protein; Table 2). We did not observe a statistically significant association between the hyperactive form of delirium and hospital mortality. After the adjusted analysis, we could also not demonstrate an independent association between any of the delirium motor subtypes and 12-month mortality (Table 3). Age, admission from the ED, malnutrition, cancer, low GFR, leukocytosis and hypoalbuminemia had statistically significant associations with decreased hospital survival. Age, admission from intensive care units, functional dependency, low GFR and elevated C-reactive protein level were predictive of higher 12-month mortality.

**Table 2:** Effects of delirium motor subtypes and covariates on hospital mortality in acutely ill older adults; 2009–2015 (N = 1,409 admissions/ 1,204 patients).

Variables	Hospital mortality N (%)	Hazard ratio, bivariate (95% CI)	Hazard ratio, adjusted ^1^ (95% CI)	Adjusted p-value
**Delirium motor subtype**				
No delirium	65 (9)	Ref.	Ref.	Ref.
Hyperactive	17 (15)	1.72 (1.01–2.94)	1.51 (0.86–2.65)	.147
Mixed	67 (34)	3.53 (2.51–4.96)	2.31 (1.53–3.50)	<.001
Hypoactive	116 (33)	3.72 (2.74–5.04)	2.43 (1.64–3.59)	<.001
**Age (10 years)**	-	1.44 (1.25–1.66)	1.25 (1.04–1.50)	.017
**Sex**				
Male	97 (18)	Ref.	Ref.	Ref.
Female	168 (20)	1.18 (0.92–1.51)	1.10 (0.81–1.50)	.530
**Marital status**				
Married	88 (16)	Ref.	Ref.	Ref.
Widowed	138 (21)	1.31 (1.01–1.72)	0.76 (0.53–1.08)	.125
Single	22 (18)	1.15 (0.72–1.84)	1.07 (0.66–1.72)	.791
Divorced/ separated	17 (19)	0.98 (0.58–1.66)	0.72 (0.39–1.32)	.288
**Referring unit**				
Outpatient clinics	79 (15)	Ref.	Ref.	Ref.
Emergency department	171 (22)	1.36 (1.04–1.78)	1.38 (1.04–1.83)	.025
Intensive care unit	15 (15)	0.95 (0.55–1.66)	0.78 (0.40–1.54)	.480
**Admission ADLs (points)**				
9–12	37 (8)	Ref.	Ref.	Ref.
5–8	43 (14)	1.67 (1.08–2.59)	1.00 (0.62–1.60)	.996
0–4	185 (30)	3.14 (2.20–4.48)	1.23 (0.79–1.90)	.358
**Mini Nutritional Assessment**				
Normal	14 (7)	Ref.	Ref.	Ref.
Risk of malnutrition	65 (11)	1.41 (0.79–2.52)	1.11 (0.56–2.19)	.758
Malnutrition	186 (31)	3.64 (2.11–6.27)	2.26 (1.14–4.48)	.020
**Polypharmacy**				
No	129 (23)	Ref.	Ref.	Ref.
Yes	136 (16)	0.76 (0.60–0.97)	0.75 (0.57–0.99)	.047
**Dementia**				
Absent	105 (15)	Ref.	Ref.	Ref.
Mild	55 (18)	1.20 (0.86–1.66)	0.85 (0.59–1.21)	.360
Moderate	34 (20)	1.33 (0.90–1.96)	0.79 (0.49–1.28)	.343
Severe	71 (35)	2.22 (1.64–3.01)	0.95 (0.64–1.42)	.819
**Cerebrovascular disease**				
No	210 (19)	Ref.	Ref.	Ref.
Yes	55 (19)	1.04 (0.78–1.41)	0.83 (0.58–1.19)	.306
**Depression**				
No	202 (18)	Ref.	Ref.	Ref.
Yes	63 (20)	1.21 (0.91–1.60)	1.20 (0.87–1.67)	.264
**Diabetes mellitus**				
No	179 (19)	Ref.	Ref.	Ref.
Yes	86 (19)	1.12 (0.87–1.45)	1.32 (0.96–1.80)	.083
**Coronary disease**				
No	218 (19)	Ref.	Ref.	Ref.
Yes	47 (19)	1.05 (0.77–1.44)	0.91 (0.61–1.35)	.637
**Heart failure**				
No	186 (18)	Ref.	Ref.	Ref.
Yes	79 (20)	1.17 (0.90–1.52)	1.31 (0.92–1.86)	.129
**COPD**				
No	227 (18)	Ref.	Ref.	Ref.
Yes	38 (22)	1.29 (0.91–1.82)	1.17 (0.81–1.70)	.395
**Cancer**				
No	212 (17)	Ref.	Ref.	Ref.
Yes	53 (34)	1.83 (1.35–2.47)	1.75 (1.22–2.50)	.002
**Infection (at admission)**				
No	110 (15)	Ref.	Ref.	Ref.
Yes	155 (23)	1.35 (1.05–1.72)	0.75 (0.57–1.00)	.049
**Heart rate (bpm)**				
60–100	230 (18)	Ref.	Ref.	Ref.
<60	8 (13)	0.58 (0.29–1.17)	0.66 (0.31–1.39)	.274
≥100	27 (27)	1.36 (0.91–2.03)	1.05 (0.66–1.65)	.849
**Mean arterial pressure (mmHg)**				
≥90	110 (16)	Ref.	Ref.	Ref.
<90	155 (22)	1.26 (0.99–1.61)	1.10 (0.85–1.43)	.476
**Albumin (g/dL)**	-	0.55 (0.46–0.67)	0.63 (0.49–0.81)	<.001
**GFR (mL/min)**				
≥60	115 (16)	Ref.	Ref.	Ref.
30–59	93 (19)	1.35 (1.03–1.78)	1.50 (1.12–1.99)	.006
<30	57 (27)	1.75 (1.27–2.41)	1.58 (1.06–2.36)	.025
**Urea (mg/dL)**				
<85	178 (16)	Ref.	Ref.	Ref.
≥85	87 (27)	1.52 (1.17–1.96)	1.10 (0.79–1.51)	.578
**Leucocytes (10**^**3**^ **cells/mm**^**3**^**)**				
<11	173 (16)	Ref.	Ref.	Ref.
≥11	92 (29)	1.70 (1.32–2.20)	1.34 (1.01–1.79)	.045
**C-reactive protein (mg/L)**				
≤50	114 (13)	Ref.	Ref.	Ref.
>50	151 (27)	1.64 (1.28–2.10)	1.20 (0.91–1.58)	.202

95% CI = confidence interval; Ref. = referent category; COPD = chronic obstructive pulmonary disease; bpm = beats per minute; GFR = glomerular filtration rate.

^1^ Cox proportional hazards model adjusted to all the variables included in the table.

**Table 3:** Effects of delirium motor subtypes and covariates on 12-month mortality in acutely ill older adults; 2009–2015 (N = 1,144 discharges/ 976 patients).

Variables	12-month mortality N (%)	Hazard ratio, bivariate (95% CI)	Hazard ratio, adjusted ^1^ (95% CI)	Adjusted p-value
**Delirium motor subtype**				
No delirium	127 (18)	Ref.	Ref.	Ref.
Hyperactive	32 (34)	2.13 (1.45–3.14)	1.37 (0.88–2.13)	.169
Mixed	35 (27)	1.54 (1.06–2.25)	0.86 (0.56–1.30)	.472
Hypoactive	78 (34)	1.99 (1.50–2.63)	0.99 (0.68–1.45)	.975
**Age (10 years)**	-	1.32 (1.15–1.53)	1.28 (1.06–1.55)	.011
**Sex**				
Male	113 (25)	Ref.	Ref.	Ref.
Female	159 (23)	0.90 (0.71–1.14)	0.92 (0.68–1.24)	.568
**Marital status**				
Married	115 (26)	Ref.	Ref.	Ref.
Widowed	126 (24)	0.95 (0.74–1.23)	0.78 (0.56–1.08)	.138
Single	15 (15)	0.53 (0.74–1.23)	0.54 (0.32–0.91)	.022
Divorced/ separated	16 (22)	0.81 (0.48–1.37)	1.01 (0.57–1.79)	.971
**Referring unit**				
Outpatient clinics	98 (21)	Ref.	Ref.	Ref.
Emergency department	140 (23)	1.11 (0.86–1.44)	0.88 (0.66–1.17)	.365
Intensive care unit	34 (40)	2.19 (1.48–3.24)	1.71 (1.07–2.75)	.026
**Admission ADLs (points)**				
9–12	60 (13)	Ref.	Ref.	Ref.
5–8	56 (21)	1.62 (1.13–2.33)	1.47 (1.01–2.14)	.042
0–4	156 (37)	3.24 (2.41–4.37)	2.39 (1.65–2.75)	<.001
**Mini Nutritional Assessment**				
Normal	33 (17)	Ref.	Ref.	Ref.
Risk of malnutrition	109 (21)	1.28 (0.86–1.88)	0.96 (0.64–1.45)	.843
Malnutrition	130 (31)	2.07 (1.41–3.03)	1.11 (0.71–1.75)	.641
**Polypharmacy**				
No	109 (25)	Ref.	Ref.	Ref.
Yes	163 (21)	0.87 (0.68–1.11)	0.93 (0.70–1.23)	.588
**Dementia**				
Absent	109 (18)	Ref.	Ref.	Ref.
Mild	62 (24)	1.40 (1.021.91)	1.05 (0.74–1.47)	.789
Moderate	40 (29)	1.77 (1.23–2.54)	1.02 (0.67–1.56)	.921
Severe	61 (46)	3.11 (2.27–4.26)	1.53 (0.98–2.39)	.059
**Cerebrovascular disease**				
No	203 (22)	Ref.	Ref.	Ref.
Yes	69 (30)	1.40 (1.06–1.84)	1.18 (0.84–1.67)	.339
**Depression**				
No	211 (24)	Ref.	Ref.	Ref.
Yes	61 (24)	1.02 (0.77–1.35)	1.11 (0.80–1.53)	.538
**Diabetes mellitus**				
No	191 (24)	Ref.	Ref.	Ref.
Yes	81 (22)	0.90 (0.69–1.17)	0.94 (0.69–1.28)	.694
**Coronary disease**				
No	235 (25)	Ref.	Ref.	Ref.
Yes	37 (19)	0.73 (0.52–1.04)	0.66 (0.44–1.00)	.052
**Heart failure**				
No	199 (24)	Ref.	Ref.	Ref.
Yes	73 (24)	0.98 (0.75–1.28)	1.21 (0.87–1.67)	.265
**COPD**				
No	239 (24)	Ref.	Ref.	Ref.
Yes	33 (25)	1.07 (0.74–1.53)	1.17 (0.79–1.73)	.426
**Cancer**				
No	247 (24)	Ref.	Ref.	Ref.
Yes	25 (25)	1.13 (0.75–1.71)	1.17 (0.72–1.90)	.534
**Infection (at admission)**				
No	121 (19)	Ref.	Ref.	Ref.
Yes	151 (30)	1.59 (1.25–2.02)	1.26 (0.95–1.67)	.107
**Heart rate (bpm)**				
60–100	240 (24)	Ref.	Ref.	Ref.
<60	11 (21)	0.85 (0.46–1.55)	0.97 (0.53–1.79)	.931
≥100	21 (29)	1.25 (0.80–1.95)	1.00 (0.61–1.63)	.991
**Mean arterial pressure (mmHg)**				
≥90	136 (23)	Ref.	Ref.	Ref.
<90	136 (24)	1.26 (0.99–1.61)	1.07 (0.84–1.37)	.583
**Albumin (g/dL)**	-	0.70 (0.58–0.84)	0.85 (0.69–1.05)	.130
**GFR (mL/min)**				
≥60	127 (22)	Ref.	Ref.	Ref.
30–59	101 (25)	1.21 (0.93–1.57)	1.50 (1.12–1.99)	.006
<30	44 (28)	1.39 (0.99–1.96)	1.58 (1.06–2.36)	.025
**Urea (mg/dL)**				
<85	210 (23)	Ref.	Ref.	Ref.
≥85	62 (26)	1.14 (0.86–1.51)	0.77 (0.55–1.08)	.125
**Leucocytes (10**^**3**^ **cells/mm**^**3**^**)**				
<11	210 (23)	Ref.	Ref.	Ref.
≥11	62 (27)	1.20 (0.91–1.60)	0.97 (0.72–1.31)	.856
**C-reactive protein (mg/L)**				
≤50	153 (21)	Ref.	Ref.	Ref.
>50	119 (29)	1.53 (1.21–1.95)	1.32 (1.02–1.70)	.033

95% CI = confidence interval; Ref. = referent category; COPD = chronic obstructive pulmonary disease; bpm = beats per minute; GFR = glomerular filtration rate.

^1^ Cox proportional hazards model adjusted to all the variables included in the table.

## Discussion

In our cohort of acutely ill hospitalized older adults, we observed that delirium occurred in 47% of admissions. One in three of these admissions resulted in death during hospitalization, with a cumulative mortality of 52% in 12 months. The high frequency of delirium and elevated mortality rates are consistent with data reported in the literature [7, 33]. We found that the predominant motor subtype of delirium was the hypoactive form (53%), and that hospitalized older adults with hypoactive delirium, either exclusively or in alternation with the hyperactive pattern, had a substantially lower hospital survival than patients without delirium or with pure hyperactive delirium. In contrast, we did not verify statistically significant associations between delirium motor subtypes and 12-month mortality. Other factors demonstrated to have prognostic importance in our cohort were age, admission from ED or ICU, functional dependency, malnutrition, cancer, reduced GFR, hypoalbuminemia, leukocytosis, and elevated C-reactive protein.

Although hypoactive delirium is usually described as potentially more severe than the other forms [34], results presented in the literature on their prognostic differences are sparse and conflicting. The discordance may result from disparities in how psychomotor activity subtypes were determined, or from differences in sample characteristics, but might also originate from methodological limitations. Kiely et al. (2007) conducted a study in eight post-acute care facilities and found that hypoactive delirium was associated with 1.6 times the rate (hazard) of 12-month mortality when compared to normal psychomotor activity [10]. The study included 457 patients with delirium and adjusted the analyses for important covariates, but their results may have been limited by the low frequency of hyperactive (10%) and mixed subtypes (12%), and by survival biases intrinsic to a study conducted in post-hospital settings. Yang et al. (2009), in a similar study environment, observed an independent association between hypoactive delirium and 6-month mortality only in older adults with dementia, with a four-fold higher risk of death [12]. In another study, Meagher et al. (2011) reported an association between hypoactive delirium and death within one month of inclusion in a cohort of 100 hospitalized patients in palliative care [13]. However, the analysis was not adjusted for possible confounding factors, and the morbidity and mortality profile of the selected population (i.e. palliative care) might have limited the external validity of the results to settings with similar goals of care.

In contrast, Marcantonio et al. (2002) reported in a sample of 122 older adults with delirium following hip fracture repair that hyperactive symptoms were associated with a six-fold increase in the odds of death or institutionalization [14]. It is noteworthy, however, that most of the cases were classified as hypoactive delirium, and that because only 2 patients presented with hyperactive delirium in its pure form, these were grouped with the cases of mixed delirium, raising concerns that the conclusion that hyperactive features of delirium were associated with increased mortality might have been over simplified. Kelly et al. (2001), DeCrane et al. (2011), and Slor et al. (2013) did not observe independent associations between delirium motor subtypes and mortality [15–17]. The first two studies analyzed the prognosis of delirium patients in nursing homes, while the third conducted a prospective study following older adults undergoing hip fracture repair. Nevertheless, interpretation of their results was limited by factors such as small sample sizes and lack of adjustments for potential confounders.

The association of hypoactive and mixed delirium subtypes with a worse prognosis was demonstrated even after adjusting the analysis for demographic, clinical and laboratory characteristics. In one of the earliest studies on delirium motor subtypes, Liptzin and Levkoff (1992) suggested that, in most cases, the expression of characteristics associated with hyperactive delirium would only happen in individuals who were sufficiently fit to manifest agitation [35]. This theory is consistent with the fact that hypoactive delirium is more common in older, frailer, more dependent patients, and therefore with the perception that it might be a marker of clinical vulnerability. The higher incidence of hypoactive delirium in long term care facilities and geriatric wards, like ours, is also consistent with this understanding [16].

It has been suggested that subtyping delirium according to altered level of arousal, which is one of the components of delirium psychomotor activity, could be simpler and easier to standardize [36]. However, it is still unclear how level of arousal relates to prognosis in patients with delirium. Han et al. (2017) observed in a cohort of 1,084 older adults from the emergency department that only delirium with normal arousal was significantly associated with increased mortality, compared to the no delirium group [37]. Further studies are needed to understand the clinical value of subtyping delirium only according to arousal level as compared to assessing additional characteristics of psychomotor activity.

Our findings on the high frequency and poor prognosis associated with hypoactive manifestations of delirium have particular relevance for clinical practice, since it is documented that physicians and nurses have greater difficulty in recognizing these cases [9]. This fact, added to the challenge of engaging the affected individuals in beneficial activities for their recovery, may impair therapeutic efficacy and subsequent clinical outcomes. Reports on the association between hypoactive delirium and other complications such as functional decline, worse quality of life, pressure ulcers and nosocomial infections, reinforce the need to expand the knowledge about the best strategies for its management [38].

Our study had limitations. It was conducted in a single center, in a unit specialized in the care of geriatric patients with high clinical complexity and vulnerability. Although many of our results are consistent with those described by other groups, these aspects may have decreased their external validity. A large proportion of patients spent more than 48 hours in the ED, which might have led to detection bias of delirium, as it could have resolved before referral to our unit. Still, the high prevalence of delirium found at admission suggests the number of cases missed in the ED is likely to have been minimum. The prolonged hospital stay and high mortality are noteworthy as well, but it is likely that these results are the product of a setting reserved for patients with the highest clinical severity, and of a health care system inefficient in providing high quality primary care and effective post-acute rehabilitation.

We sought with this study to provide new evidence to a field that still lacks consensus information. To this end, we examined the association of delirium motor subtypes with mortality, adjusting our analyses for numerous clinical variables that have not yet been simultaneously controlled for in this context. Nevertheless, delirium is a complex and multifaceted syndrome, and it is reasonable to assume that there are predictors of mortality in this population that were not measured or contemplated. Among these factors, the duration of delirium episodes is one aspect that has been indicated as a prognostic marker and was not investigated in our work [11]. The interface between delirium motor subtypes and delirium severity also needs to be further explored. Even though Delirium Index contemplates drowsiness and unresponsiveness in several of its items, only one of them is specific to motor disturbances, therefore assessments that provided additional granularity to this element would be important.

Finally, categorizing delirium by its psychomotor subtypes can be complex and, despite using CAM guidelines to characterize psychomotor agitation and/or retardation, we did not formally apply an existing instrument to classify the different delirium subtypes, such as the Delirium Motor Subtype Scale [5]. The involvement of multiple geriatric fellows as study examiners performing geriatric assessments may also have affected the reliability of the measures, although they were routinely trained to ensure the consistency of the method. Previous studies have reported a substantial proportion of delirious patients with normal psychomotor activity [12]. While this has not been our experience and that of other groups [39], we must accept the possibility that cases of delirium without motor disturbances might have been misclassified. Nonetheless, we believe that due to the exuberant nature of the hyperactive symptoms of delirium, it is unlikely that cases with normal psychomotor activity would have been misclassified as having hyperactive delirium. On the other hand, while there could be a greater risk of categorizing normal motor subtype of delirium as hypoactive delirium, this would probably have pulled our results towards the null [10], meaning that the associations we found would in fact be even greater.

The study also had many positive aspects. This is one of the largest cohorts ever to be analyzed investigating the effects of delirium motor subtypes on the mortality of acutely ill older adults. The sample size provided adequate power to detect statistically significant associations and allowed us to adjust the multivariate analysis models for various relevant characteristics. Data were recorded prospectively and systematically, following a standardized model of comprehensive geriatric assessment, which allowed for a detailed view of the health of the patients admitted to our unit. After discharge, loss to follow-up was under 5% and non-differential between non-delirium and delirium groups, minimizing this issue as a potential source of bias. Lastly, the ward routines ensured a horizontal follow-up of its clientele, with supervision by experienced geriatricians and daily discussions on all the admissions, which reduced the risk of non-detection of delirium and other geriatric syndromes.

Characterization of delirium motor subtypes is a potentially important element to identify patients at high risk for poor clinical outcomes. This type of evaluation can assist in the selection of candidates for targeted therapeutic interventions, resource allocation, and decisions on advanced life support. The possibility of stratifying risk can be useful for health professionals who need to help patients and families better understand the clinical recovery process and likely outcomes [7]. Our study indicates that the occurrence of delirium in its hypoactive and mixed subtypes should be used in the prediction of hospital mortality in acutely ill older adults. Still, the attending staff must be attentive to the importance of other clinical characteristics to estimate short and/or long-term survival. Future studies should focus on the use of motor subtypes to refine measures of delirium severity, and on individualized strategies to improve clinical outcomes.

## Supporting information

S1 Table Demographic characteristics of acutely ill hospitalized older adults, according to delirium motor subtype; 2009–2015. (DOCX)

S2 Table Geriatric syndromes and comorbidities in acutely ill hospitalized older adults, according to delirium motor subtype; 2009–2015. (DOCX)

S3 Table Vital signs and laboratory tests at admission of acutely ill older adults, according to delirium motor subtype; 2009–2015. (DOCX)
