# Supplementary material for: Prognostic effects of delirium motor subtypes in hospitalized older adults: A prospective cohort study
Source: PLoS One. 2018 Jan 30;13(1):e0191092. doi: 10.1371/journal.pone.0191092 (PMC5790217; doi:10.1371/journal.pone.0191092)
Supplement: S1 Table — (DOCX) [file pone.0191092.s001.docx]

**S1 Table. Demographic characteristics of acutely ill hospitalized older adults, according to delirium motor subtype; 2009-2015.**

| **Characteristics, N (%)** | **Total**  **1409 (100)** | **No delirium**  **752 (53)** | **Hyperactive delirium**  **112 (8)** | **Mixed delirium**  **197 (14)** | **Hypoactive delirium**  **348 (25)** | **Pearson**  ***X*^2^** | ***One-way ANOVA F*** | **p-value** |
| --- | --- | --- | --- | --- | --- | --- | --- | --- |
| Age (years), mean (SD) | 80 (±9) | 79 (±9) | 81 (±8) | 83 (±9) | 83 (±9) | - | 25 | <.001 |
| 60-69 | 175 (12) | 127 (17) | 9 (8) | 15 (8) | 24 (7) | 66 | - | <.001 |
| 70-79 | 440 (31) | 264 (35) | 38 (34) | 49 (25) | 89 (26) |  |  |  |
| 80-89 | 558 (40) | 267 (36) | 45 (40) | 81 (41) | 165 (47) |  |  |  |
| ≥90 | 236 (17) | 94 (13) | 20 (18) | 52 (26) | 70 (20) |  |  |  |
| Female | 860 (61) | 451 (60) | 57 (51) | 116 (59) | 236 (68) | 12 | - | .006 |
| Race |  |  |  |  |  |  |  |  |
| White | 945 (67) | 493 (66) | 76 (68) | 130 (66) | 246 (71) | 12 | - | .195 |
| Mixed | 244 (17) | 126 (17) | 24 (21) | 37 (19) | 57 (16) |  |  |  |
| Black | 149 (11) | 92 (12) | 7 (6) | 16 (8) | 34 (10) |  |  |  |
| Other | 71 (5) | 41 (5) | 5 (4) | 14 (7) | 11 (3) |  |  |  |
| Marital status |  |  |  |  |  |  |  |  |
| Married | 539 (38) | 313 (41) | 51 (46) | 66 (34) | 109 (31) | * | - | <.001 |
| Widowed | 657 (47) | 322 (43) | 40 (36) | 101 (51) | 194 (56) |  |  |  |
| Single | 124 (9) | 62 (8) | 18 (16) | 15 (8) | 29 (8) |  |  |  |
| Divorced/ separated | 89 (6) | 55 (7) | 3 (3) | 15 (8) | 16 (5) |  |  |  |
| Years of education |  |  |  |  |  |  |  |  |
| ≥8 | 377 (27) | 205 (27) | 29 (26) | 45 (23) | 98 (28) | 4 | - | .712 |
| 4-7 | 541 (38) | 390 (39) | 38 (34) | 82 (42) | 119 (34) |  |  |  |
| <4 | 491 (35) | 257 (34) | 45 (40) | 70 (36) | 119 (34) |  |  |  |
| Economic classification ^1^ |  |  |  |  |  |  |  |  |
| A (35-46) | 39 (3) | 16 (2) | 3 (3) | 7 (4) | 13 (4) | * | - | .517 |
| B (23-34) | 480 (34) | 252 (34) | 33 (29) | 67 (34) | 128 (37) |  |  |  |
| C (14-22) | 735 (52) | 403 (54) | 60 (54) | 97 (49) | 175 (50) |  |  |  |
| D/E (0-13) | 155 (11) | 81 (11) | 16 (14) | 26 (13) | 32 (9) |  |  |  |
| Referring unit |  |  |  |  |  |  |  |  |
| Outpatient clinics | 536 (38) | 358 (48) | 24 (21) | 58 (29) | 96 (28) | 86 | - | <.001 |
| Emergency department | 772 (55) | 351 (47) | 72 (64) | 128 (65) | 221 (64) |  |  |  |
| Intensive care | 101 (7) | 43 (6) | 16 (14) | 11 (6) | 31 (9) |  |  |  |

SD= standard deviations.

^1^ Economic classification scores range from 0 to 46, higher scores meaning better economic conditions.

* Fisher’s exact test was used.
