# Supplementary material for: Prognostic effects of delirium motor subtypes in hospitalized older adults: A prospective cohort study
Source: PLoS One. 2018 Jan 30;13(1):e0191092. doi: 10.1371/journal.pone.0191092 (PMC5790217; doi:10.1371/journal.pone.0191092)
Supplement: S2 Table — (DOCX) [file pone.0191092.s002.docx]

S2 Table. Geriatric syndromes and comorbidities in acutely ill hospitalized older adults, according to delirium motor subtype; 2009-2015.

| **Characteristics, N (%)** | **Total**  **1409 (100)** | **No delirium**  **752 (53)** | **Hyperactive delirium**  **112 (8)** | **Mixed delirium**  **197 (14)** | **Hypoactive delirium**  **348 (25)** | **Pearson**  ***X*^2^** | **p-value** |
| --- | --- | --- | --- | --- | --- | --- | --- |
| **Geriatric assessment** |  |  |  |  |  |  |  |
| Polypharmacy | 851 (60) | 467 (62) | 65 (58) | 109 (55) | 210 (60) | 3 | .349 |
| Pre-admission ADLs (points) |  |  |  |  |  |  |  |
| 9-12 | 929 (66) | 578 (77) | 60 (54) | 116 (59) | 175 (50) | 98 | <.001 |
| 5-8 | 238 (17) | 101 (13) | 26 (23) | 35 (18) | 76 (22) |  |  |
| 0-4 | 242 (17) | 73 (10) | 26 (23) | 46 (23) | 97 (28) |  |  |
| Admission ADLs (points) |  |  |  |  |  |  |  |
| 9-12 | 485 (34) | 402 (53) | 22 (20) | 26 (13) | 35 (10) | 353 | <.001 |
| 5-8 | 315 (22) | 187 (25) | 24 (21) | 36 (18) | 68 (20) |  |  |
| 0-4 | 609 (43) | 163 (22) | 66 (59) | 135 (69) | 245 (70) |  |  |
| Depression | 313 (22) | 162 (22) | 15 (15) | 52 (26) | 82 (24) | 6 | .124 |
| Dementia |  |  |  |  |  |  |  |
| Absent | 722 (51) | 504 (67) | 41 (37) | 75 (38) | 102 (29) | 201 | <.001 |
| Mild | 312 (22) | 142 (19) | 24 (21) | 59 (30) | 87 (25) |  |  |
| Moderate | 171 (12) | 56 (7) | 25 (22) | 25 (13) | 65 (19) |  |  |
| Severe | 204 (14) | 50 (7) | 22 (20) | 38 (19) | 94 (27) |  |  |
| Urinary incontinence | 848 (60) | 404 (54) | 65 (58) | 133 (68) | 246 (71) | 34 | <.001 |
| Two or more falls (12 months) | 175 (12) | 67 (9) | 19 (17) | 39 (20) | 50 (14) | 22 | <.001 |
| Visual impairment | 663 (47) | 350 (47) | 43 (38) | 103 (52) | 167 (48) | 6 | .125 |
| Hearing impairment | 416 (29) | 214 (28) | 29 (26) | 66 (34) | 107 (31) | 3 | .412 |
| Pressure ulcers |  |  |  |  |  |  |  |
| None | 1121 (80) | 632 (84) | 87 (78) | 161 (82) | 241 (69) | 35 | <.001 |
| One | 180 (13) | 76 (10) | 18 (16) | 23 (12) | 63 (18) |  |  |
| Two or more | 108 (8) | 44 (6) | 7 (6) | 13 (7) | 44 (13) |  |  |
| Mini Nutritional Assessment |  |  |  |  |  |  |  |
| Normal (≥12) | 213 (15) | 166 (22) | 13 (12) | 13 (7) | 21 (6) | 79 | <.001 |
| Risk of malnutrition (8-11) | 595 (42) | 362 (48) | 58 (52) | 70 (36) | 105 (30) |  |  |
| Malnutrition (≤7) | 601 (43) | 224 (30) | 41 (37) | 114 (58) | 222 (64) |  |  |
| **Comorbidities** |  |  |  |  |  |  |  |
| Hypertension | 1020 (72) | 546 (73) | 85 (76) | 141 (72) | 248 (71) | 1 | .803 |
| Diabetes | 448 (32) | 229 (30) | 39 (35) | 68 (35) | 112 (32) | 2 | .616 |
| Heart failure | 389 (28) | 216 (29) | 28 (25) | 59 (30) | 86 (25) | 3 | .415 |
| Previous stroke | 286 (20) | 123 (16) | 24 (21) | 46 (23) | 93 (27) | 17 | .001 |
| Coronary disease | 246 (17) | 138 (18) | 15 (13) | 32 (16) | 61 (18) | 2 | .593 |
| COPD | 172 (12) | 100 (13) | 12 (11) | 20 (10) | 40 (11) | 2 | .571 |
| Cancer | 154 (11) | 86 (11) | 10 (9) | 23 (12) | 35 (10) | 3 | .791 |
| Charlson ≥4 points | 565 (40) | 269 (36) | 44 (39) | 94 (48) | 158 (45) | 15 | .002 |

ADLs= activities of daily living; COPD= chronic obstructive pulmonary disease; Charlson= Charlson Comorbidity Index.
