# Supplementary material for: Prognostic effects of delirium motor subtypes in hospitalized older adults: A prospective cohort study
Source: PLoS One. 2018 Jan 30;13(1):e0191092. doi: 10.1371/journal.pone.0191092 (PMC5790217; doi:10.1371/journal.pone.0191092)
Supplement: S3 Table — (DOCX) [file pone.0191092.s003.docx]

S3 Table. Vital signs and laboratory tests at admission of acutely ill older adults, according to delirium motor subtype; 2009-2015.

| **Characteristics, N (%)** | **Total**  **1409 (100)** | **No delirium**  **752 (53)** | **Hyperactive delirium**  **112 (8)** | **Mixed delirium**  **197 (14)** | **Hypoactive delirium**  **348 (25)** | **Pearson**  ***X*^2^** | **p-value** |
| --- | --- | --- | --- | --- | --- | --- | --- |
| **Vital signs** |  |  |  |  |  |  |  |
| Heart rate (bpm) |  |  |  |  |  |  |  |
| <60 | 61 (4) | 38 (5) | 5 (5) | 5 (3) | 13 (4) | 6 | .434 |
| 60-100 | 1248 (89) | 669 (89) | 99 (88) | 174 (88) | 306 (88) |  |  |
| ≥100 | 100 (7) | 45 (6) | 8 (7) | 18 (9) | 29 (8) |  |  |
| Mean arterial pressure <90mmHg | 719 (51) | 380 (51) | 52 (46) | 106 (54) | 181 (52) | 2 | .622 |
| Level of consciousness |  |  |  |  |  |  |  |
| Glasgow ≥14 | 1085 (77) | 696 (93) | 77 (69) | 128 (65) | 184 (53) | 238 | <.001 |
| Glasgow 11-13 | 246 (17) | 41 (5) | 27 (24) | 54 (27) | 124 (36) |  |  |
| Glasgow <11 | 78 (6) | 15 (2) | 8 (7) | 15 (8) | 40 (11) |  |  |
| **Laboratory tests** |  |  |  |  |  |  |  |
| Hemoglobin ≤10 g/dL | 466 (33) | 241 (32) | 31 (28) | 67 (34) | 127 (36) | 4 | .290 |
| Leucocytes ≥11*10^3^ cells/mm^3^ | 321 (23) | 143 (19) | 26 (23) | 60 (30) | 92 (26) | 15 | .002 |
| Albumin <3.3 g/dL | 843 (60) | 393 (52) | 77 (69) | 126 (64) | 247 (71) | 41 | <.001 |
| Vitamin D (ng/mL) |  |  |  |  |  |  |  |
| ≥20 | 573 (41) | 312 (41) | 38 (34) | 74 (38) | 149 (43) | 10 | .113 |
| 10-19 | 362 (26) | 197 (26) | 25 (22) | 61 (31) | 79 (23) |  |  |
| <10 | 474 (34) | 243 (32) | 49 (44) | 62 (31) | 120 (34) |  |  |
| GFR (mL/min) |  |  |  |  |  |  |  |
| ≥60 | 705 (50) | 382 (51) | 70 (63) | 89 (45) | 164 (47) | 27 | <.001 |
| 30-59 | 492 (35) | 275 (36) | 28 (25) | 59 (30) | 130 (37) |  |  |
| <30 | 212 (15) | 95 (13) | 14 (13) | 49 (25) | 54 (16) |  |  |
| Urea ≥85 mg/dL | 328 (23) | 149 (20) | 17 (15) | 63 (32) | 99 (28) | 23 | <.001 |
| Sodium (mEq/L) |  |  |  |  |  |  |  |
| <125 | 30 (2) | 16 (2) | 1 (1) | 4 (2) | 9 (3) | * | <.001 |
| 125-144 | 1128 (80) | 636 (85) | 88 (79) | 149 (76) | 255 (73) |  |  |
| ≥145 | 251 (18) | 100 (13) | 23 (21) | 44 (22) | 84 (24) |  |  |
| Potassium (mEq/L) |  |  |  |  |  |  |  |
| <3.5 | 184 (13) | 86 (11) | 12 (11) | 25 (13) | 61 (18) | * | .097 |
| 3.5-5.4 | 1181 (84) | 644 (86) | 98 (88) | 163 (83) | 276 (79) |  |  |
| ≥5.5 | 44 (3) | 22 (3) | 2 (2) | 9 (5) | 11 (3) |  |  |
| Bicarbonate <20 mmol/L | 163 (12) | 82 (11) | 17 (15) | 25 (13) | 39 (11) | 2 | .565 |
| C-reactive protein >50 mg/L | 560 (40) | 254 (34) | 51 (46) | 94 (48) | 161 (46) | 24 | <.001 |

Glasgow= Glasgow Coma Scale; bpm= beats per minute; GFR= glomerular filtration rate.

* Fisher’s exact test was used.
